# Genome-wide gene copy number and expression analysis of primary gastric tumors and gastric cancer cell lines

**DOI:** 10.1186/1471-2407-10-73

**Published:** 2010-03-01

**Authors:** Siina Junnila, Arto Kokkola, Marja-Liisa Karjalainen-Lindsberg, Pauli Puolakkainen, Outi Monni

**Affiliations:** 1 Institute of Biomedicine, Medical Biochemistry and Developmental Biology, Genome-Scale Biology Research Program, University of Helsinki, Helsinki, Finland; 2 Department of Surgery, Helsinki University Central Hospital, University of Helsinki, Helsinki, Finland; 3 Department of Pathology, Haartman Institute and HUSLAB, University of Helsinki and Helsinki University Central Hospital, Helsinki, Finland; 4 Department of Surgery, Turku University Hospital, Turku, Finland

## Abstract

**Background:**

Gastric cancer is one of the most common malignancies worldwide and the second most common cause of cancer related death. Gene copy number alterations play an important role in the development of gastric cancer and a change in gene copy number is one of the main mechanisms for a cancer cell to control the expression of potential oncogenes and tumor suppressor genes.

**Methods:**

To highlight genes of potential biological and clinical relevance in gastric cancer, we carried out a systematic array-based survey of gene expression and copy number levels in primary gastric tumors and gastric cancer cell lines and validated the results using an affinity capture based transcript analysis (TRAC assay) and real-time qRT-PCR.

**Results:**

Integrated microarray analysis revealed altogether 256 genes that were located in recurrent regions of gains or losses and had at least a 2-fold copy number- associated change in their gene expression. The expression levels of 13 of these genes, *ALPK2*, *ASAP1, CEACAM5*, *CYP3A4, ENAH*, *ERBB2*, *HHIPL2*, *LTB4R*, *MMP9*, *PERLD1*, *PNMT*, *PTPRA*, and *OSMR*, were validated in a total of 118 gastric samples using either the qRT-PCR or TRAC assay. All of these 13 genes were differentially expressed between cancerous samples and nonmalignant tissues (p < 0.05) and the association between copy number and gene expression changes was validated for nine (69.2%) of these genes (p < 0.05).

**Conclusion:**

In conclusion, integrated gene expression and copy number microarray analysis highlighted genes that may be critically important for gastric carcinogenesis. TRAC and qRT-PCR analyses validated the microarray results and therefore the role of these genes as potential biomarkers for gastric cancer.

## Background

Due to the lack of early symptoms gastric adenocarcinoma is characterized by late stage diagnosis and unsatisfactory options for curative treatment [1,2]. Despite the decline in its incidence in the past few decades, gastric cancer remains the second most common cause of cancer-related deaths worldwide [3]. Approximately 90% of all gastric cancers are adenocarcinomas arising from the epithelium [4]. According to Laurén's classification gastric cancers are divided into two main histological subtypes, intestinal and diffuse [5].

Gastric adenocarcinomas, like many other solid tumors of epithelial origin, are often complex in terms of chromosomal integrity [6,7]. Malignant gastric tumors are known to carry multiple aberrations in their genome and such chromosomal alterations are crucial for the activation and inactivation of cancer-related genes [8-17]. Gene copy number change is one of the main mechanisms for a cancer cell to control the expression of genes pivotal to cell survival and cancer progression [17-22]. These copy number alterations often involve a large group of genes located close to one another in the same chromosome. For example; in gastric cancers the frequently amplified 17q12-q21 region contains genes such as *ERBB2*, *GRB7*, *JUP*, *PERLD1, PNMT*, *PPP1R1B*, *STARD3*, and *TOP2A *[14,17,23]. However, only a minority of these genes are likely to be the true cancer driver genes contributing to tumorigenesis, while others may be amplified simply because of their chromosomal proximity with the amplification target genes [24,25]. One approach to distinguish such driver genes from the passenger mutations is to integrate genome-wide copy number and expression data, which enables the identification of genes whose transcriptional activation or repression is associated with a copy number change in a cancer cell. Thus, by combining information from the high resolution gene copy number and expression microarrays, it is possible not only to define breakpoints of copy number changes in great detail, but also to assess the functional significance of these changes and therefore possibly identify genes that drive cancer onset and progression.

To highlight genes potential as biomarkers or clinical targets in gastric cancer, we carried out a systematic high-resolution array-based survey of copy number and gene expression levels in gastric cancer tissues and cell lines. Our previous array-based analysis showed that copy number gains and losses of hundreds of genes are associated with a simultaneous increase or decrease in gene expression [17]. In the present study, we have increased the resolution of the copy number analysis over 20-fold to more accurately visualize the breakpoints of the copy number alterations. Furthermore, we have carried out a transcriptional analysis of genes located in altered chromosomal regions to identify genes whose deregulation is associated with the malignant phenotype.

## Methods

### Gastric cancer tissues and cell lines

This research project has been reviewed and approved by the Ethical Committee of the Department of Medical Genetics and Surgery and authorized by the Clinical Review Board of Helsinki University Central Hospital. Gastric tissue samples were prospectively collected from patients who underwent gastric surgery or gastroscopy in the Helsinki University Central Hospital between 1999 and 2007. Informed consent was obtained from each participating patient. Thirteen fresh frozen primary gastric cancer tissues and seven gastric cancer cell lines were chosen for microarray analysis (Table 1). The tissue material consisted of two different histological subtypes, intestinal (n = 9) and diffuse (n = 4) and the tumors were located at two different sites of the stomach, the corpus (n = 8) and the antrum (n = 5). Altogether 111 gastric tissues and 7 gastric cancer cell lines were included in the qRT-PCR and the affinity capture based transcript assay (TRAC) analyses (Additional file 1: Clinical parameters). The tissue samples consisted of 43 nonmalignant and 68 cancerous gastric tissues and both histological subtypes of gastric cancer were represented (intestinal, n = 42; diffuse, n = 25; one of unknown histology). Gastric tissue samples were stored at -80°C. To verify the tumor percentage and histology of the samples, frozen samples were embedded in Tissue-Tek OCT Compound (Sakura Finetek, Torrance, CA, USA) and 5 μm frozen ice-sections were prepared and stained using Trypan Blue. Histology of gastric cancer specimens was evaluated by an experienced pathologist (M.-L. K.-L.). Tissue-Tek was removed from the tissues prior to nucleic acid extractions.

**Table 1:** Clinical parameters for the samples analyzed on array comparative genomic hybridization (aCGH) and expression microarrays.

Primary gastric tumors	Age/sex	Histology	Location
14TA	58/M	Intestinal	Corpus
200A	57/F	Intestinal	Corpus
222A	50/M	Intestinal	Corpus
232A	83/M	Intestinal	Corpus
3TC	57/F	Intestinal	Corpus
4T/N	72/M	Intestinal	Corpus
10TB	59/M	Intestinal	Antrum
17TA	77/M	Intestinal	Antrum
185B	78/F	Intestinal	Antrum
1AT/N	41/F	Diffuse	Corpus
6TB	77/F	Diffuse	Corpus
9TD	74/F	Diffuse	Antrum
13TA	56/F	Diffuse	Antrum

**Gastric cancer cell lines**	**Age/sex**	**Histology**	**Origin**

AGS	54/F	Adeno-carcinoma	Primary tumor
KATOIII	55/M	Diffuse	Pleural effusion
MKN-1	72/M	Adeno-squamous carcinoma	Lymph node metastasis
MKN-7	39/M	Intestinal	Lymph node metastasis
MKN-28	70/F	Intestinal	Lymph node metastasis
MKN-45	62/F	Diffuse	Liver metastasis
TMK-1	21/M	Diffuse	Lymph node metastasis

AGS and KATOIII cell lines were obtained from American Type Culture Collection (Rockville, MD, USA) and MKN-1, MKN-7, MKN-28, MKN-45, and TMK-1 cell lines were a kind gift from Hiroshi Yokozaki, Kobe University Graduate School of Medicine, Kobe, Japan [26]. AGS cells were grown in Kaighn's F12 medium (2 mM glutamine, 10% FBS, 100 U/ml penicillin-streptomycin), KATOIII cells in IMDM medium (2 mM glutamine, 10% FBS, 100 U/ml penicillin-streptomycin) and all other cell lines in RPMI-1640 medium (10% FCS, 2 mM glutamine, 100 U/ml penicillin-streptomycin). All cells were grown at 37°C and 5% CO_2_.

### RNA and DNA extraction

Prior to RNA and DNA extractions, the frozen tissue was immersed in RNAlater-ICE reagent (Ambion, Austin, TX, USA) and stored at -80°C for 16 hours to stabilize the RNA. Half of the tissue sample (~ 25 mg) was homogenized in RLT-β-mercaptoethanol lysis buffer (RNeasy mini kit, Qiagen Inc., Hilden, Germany) and the other half in ATL-buffer (DNeasy Blood and Tissue Kit, Qiagen) using the Ultra-Turrax homogenizer (IKA Works, Wilmington, NC, USA). RNA was extracted using the RNeasy mini kit, including the optional DNase treatment, and DNA using the DNeasy Blood and Tissue Kit. For gastric cancer cell lines, 1 × 10^7 ^cells were lysed using a syringe and needle in either RLT-β-mercaptoethanol lysis buffer or ATL-buffer prior to RNA and DNA extractions, respectively. RNA and DNA concentrations were measured using NanoDrop1000 (Thermo Fisher Scientific, Waltham, MA, USA) and RNA quality was evaluated using Agilent's 2100 Bioanalyzer (Agilent Technologies, Palo Alto, CA, USA). Only RNAs showing distinct 18S and 28S ribosomal peaks in the Bioanalyzer analysis and 260/280 ratios above 2.0 were accepted for further analysis.

### Array CGH and gene expression microarray analyses

**Figure 1:**
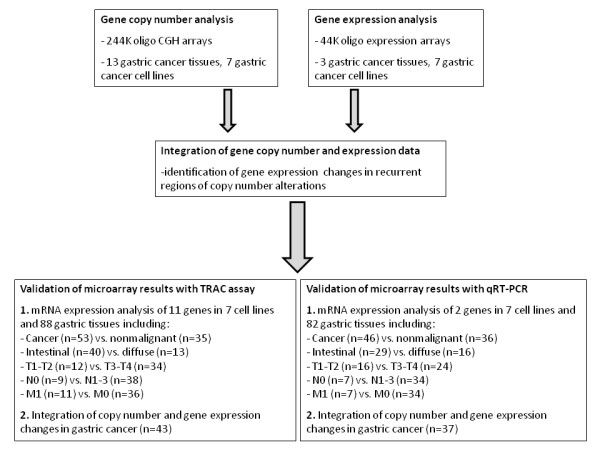
**Flowchart describing different steps of the study**.

Thirteen gastric tumors and seven gastric cancer cell lines were analyzed on the 244K Human Genome CGH oligoarrays (G4411B, Agilent Technologies). Three of the tumors and all of the seven cell lines were also analyzed using the 44K Whole Human Genome gene expression oligoarrays (G4112F, Agilent Technologies) (Figure 1). The mean 260/280 ratios for these samples were 2.1 for RNA and 1.8 for DNA, and all of the RNA samples had clear 18S and 28S ribosomal peaks in the Bioanalyzer analysis indicating good quality (data not shown). Array CGH experiments were performed using Human Genome CGH Microarray 244A kit (Agilent Technologies). Labeling and hybridization were performed according to the Agilent's protocol (v5.0, June 2007). In brief, 1.5 μg of sample DNA and 1.5 μg of sex-matched reference DNA (Human Genomic DNA, Promega, Madison, WI, USA) were double-digested with *AluI *and *RsaI *restriction enzymes (Promega). The digested DNA was labeled using the Agilent Genomic DNA Labeling Kit Plus. Sample DNA was labeled with Cy5-dUTP and reference DNA with Cy3-dUTP, respectively. Labeled DNA was purified with Microcon YM-30 filters (Millipore, Billerica, MA, USA). Following the purification, sample and reference DNAs were pooled and hybridized to the array with 50 μg of Human Cot-1 DNA (Invitrogen, Carlsbad, CA, USA) at 65°C, 20 rpm for 40 h. Hybridization was performed with Agilent Oligo aCGH Hybridization Kit. Prior to scanning, the slides were washed according to the protocol. In addition to the sample DNA hybridizations described above, reference male DNA (Cy3) was hybridized against reference female DNA (Cy5) according to the same protocol to be used as a reference array in the aCGH data analysis.

Gene expression experiments were performed using the Whole Human Genome Oligo Microarray kit (Agilent Technologies), and labeling and hybridization according to the Agilent protocol (v5.7, March 2008). In brief, 2 μg of total sample RNA and reference RNA (a pool of 10 cancer cell lines, non-gastric, ATCC, Manassas, MA, USA) were labeled using the Agilent Quick Amp Labeling Kit. Sample RNA was labeled with Cy5-dCTP and reference DNA with Cy3-dCTP, respectively. Labeled RNA was then purified using RNeasy mini spin columns (Qiagen). Hybridization was performed with Agilent Gene Expression Hybridization Kit and samples were hybridized at 65°C, 10 rpm for 17 h and washed according to the protocol prior to scanning. Both aCGH and gene expression microarray slides were scanned using the DNA Microarray Scanner (Agilent Technologies) and analyzed with Feature Extraction Software (v9.5.1.1.).

### High-resolution copy number profiling

All copy number data is available at http://www.cangem.org (accession number: CG-EXP-49) [27]. Agilent's CGH Analytics software (v3.5.14) was applied to identify the copy number changes. Microarray data was quality filtered using the outlier information obtained from the Feature Extraction analysis. Probes flagged as outliers were removed from further analysis. In addition, the following aberration filters were applied: minimum number of probes in region = 3, minimum absolute log_2 _ratio for region = 0.27, and maximum number of aberrant regions = 1000. The log_2 _ratio of 0.27 corresponds to a 1.2-fold change in the copy number. In CGH Analytics, each aCGH ratio was first converted to a log_2 _ratio followed by a Z-normalization. The male vs. female reference array was used as a calibration array in the data analysis. Because of the gender differences between the arrays that could cause bias in the analysis, chromosomes X and Y were excluded from the calibration. ADM-2 algorithm with a threshold level of 12.0 was used to identify gene copy number alterations in individual samples and cell lines. Minimal common regions of alterations in the 20 samples were calculated, including the size and chromosomal position of the alteration in base pairs. An aberration was defined as recurrent, if it was present in at least 25% of the samples (Table 2).

**Table 2:** Minimal common regions of recurrent (≥25%) copy number alterations.

Alteration	Tissues (n = 13)	Cell lines (n = 7)	Frequency	Size (Mb)	Position (Mb)	Possible target genes
+1q41-q43.1	2	3	25%	17.30	216.31-233.61	*ENAH, AGT, CAPN2, LEFTY2, LGALS8*
+5p13.3-q11.1	1	4	25%	19.41	30.18-49.60	*OSMR, RNASEN*
+7q21.3-q22.1	4	3	35%	4.60	97.33-101.93	*CYP3A4, AZGP1, VGF*
+8q24.13-q24.3	3	2	25%	19.8	126.45-146.25	*ASAP1, BAI1, KHDRBS3*
+8q24.3	6	3	45%	2.23	143.59-145.82	*GML, LYPD, AK3*
**+**14q11.2	0	5	25%	1.05	22.89-23.94	*LTB4R*
+17q12-q21.1	3	3	30%	0.28	35.02-35.30	*ERBB2, PPP1R1B, PERLD1, PNMT*
+17q22-q24.2	2	3	25%	13.65	50.45-64.10	*AXIN2, RNF43*
+19q12-qter	4	3	35%	29.36	33.89-63.25	*CEACAM5, APOC1, APOE, CEACAM7, FTL, FUT1, GPR4, HPN, KCNN4, KLK1, KLK12, LYPD3, NLRP7, CCNE1*
+20p13-qter	5	3	40%	57.94	0.04-57.98	*PTPRA, BLCAP, CD40, CHGB, CST3, EYA2, PI3, ID1, MMP9, BMP7*
-9p24.3-p21.1	3	4	35%	27.81	1.05-28.86	*MTAP, CD274, INSL4, JAK2, MLANA, SMARC2, TUSC1*
-18q12.3-q22.2	3	5	40%	26.11	39.48-65.59	*SMAD7, SERPINB2/B3/B4/B5*
-18q22.3-qter	2	5	35%	3.69	70.95-74.65	*TSHZ1*
-21q11.2-q21.1	3	3	30%	4.07	14.37-19.44	*HSPA13*
-Xq28	4	1	25%	1.21	152.24-153.45	*-*

Number of cases, size of the minimal common regions (Mb), and the chromosomal position of the alteration (Mb) are indicated as well as possible target genes. CNV regions are not shown in the table. Copy number gain (+), copy number loss (-).

### Gene expression microarray analysis

All gene expression data is available at http://www.cangem.org (accession number: CG-EXP-49) [27]. Microarray results were quality filtered using outliers defined by the Feature Extraction Software and normalized according to the Loess method, which was included in the software package. The gene expression analysis was restricted to genes located in the chromosomal regions with recurrent aberrations (Table 2). The goal of this approach was to highlight gene expression changes that were associated with changes in the gene copy number, and could therefore represent potential oncogenes or tumor suppressor genes with a functional role in cancer. First, a median log10 expression ratio was calculated for all the probes targeting the same gene. Then, in two separate analyses for gains and losses, the median expression level of each gene was compared between the samples with copy number gain/loss and samples with normal copy number to evaluate the effect of copy number alterations on gene expression. Gene expression fold changes (FC) were calculated either by dividing the median expression of the cancerous samples by the median expression of the nonmalignant samples or by dividing the median expression of cancer samples with copy number alterations (g1) by the median expression of cancer samples with normal copy number (g0). At least a 2-fold copy number associated change in gene expression was considered significant. Based on this data, 13 genes *ALPK2*, *ASAP1*, *CEACAM5*, *CYP3A4*, *ENAH*, *ERBB2, HHIPL2, LTB4R*, *MMP9, OSMR*, *PERLD1*, *PNMT*, and *PTPRA*, were chosen to be further validated with qRT-PCR analysis and TRAC (transcript analysis with aid of affinity capture) assay. The results from the integrated microarray analysis were compared with three previously published studies that systematically integrate genome-wide copy number and gene expression data [15-17].

### Real-time qRT-PCR analysis

Real-time qRT-PCR was performed for 2 genes, *ALPK2 *(18q21.31-q21.32) and *HHIPL2 *(1q41). The expression levels were measured in 82 gastric tissues (46 cancerous and 36 nonmalignant tissues) and in 7 gastric cancer cell lines (Additional file 1: Clinical parameters). 1 μg of total RNA was converted to cDNA using Moloney-murine leukemia virus reverse transcriptase (Promega, Madison, WI, USA) and random primers (Invitrogen) in a volume of 50 μl for 1 h at 37°C. The reaction was heat-inactivated (95°C, 3 min) and filled to a final volume of 200 μl with molecular grade water. The transcripts were quantitated using the Assays-on-DemandTM gene expression products (Hs01085414_m1 for *ALPK2 *and Hs00226924_m1 for *HHIPL2*) according to the manufacturer's protocol (Applied Biosystems, Foster City, CA, USA). All primers were located on exon-exon boundaries. Briefly, 2 μl of cDNA template was mixed with 1.25 μl of specific primers and probes labeled with FAM-reporter dye. 12.5 μl of TaqMan^® ^Universal PCR Mastermix and RNase-free water were added to a total volume of 25 μl. Human 18S rRNA served as an endogenous control to normalize the expression levels in the subsequent quantitative analysis. The 18S probe was labeled with VIC-reporter dye to allow multiplex PCR with the target genes. The PCR conditions were as follows: 50°C for 2 min, 95°C for 10 min, followed by 40 cycles of 95°C for 15 s and 60°C for 1 min. Each sample was measured in triplicate and the data were analyzed by the delta-delta method for comparing relative expression results (2^-[Ct sample-Ct control]^).

### TRAC assay

Transcript analysis with aid of affinity capture (TRAC) assay [28] was performed for 11 different genes in 88 gastric tissues (53 cancerous and 35 nonmalignant tissues) and 7 gastric cancer cell lines (Additional file 1: Clinical parameters). The genes included in the analysis were *ENAH *(1q42.12), *OSMR *(5p13.1), *CYP3A4 *(7q21.1) *ASAP1 *(8q24.1-q24.2), *LTB4R *(14q11.2-q12), *PERLD1 *(17q12), *ERBB2 *(17q21.1), *PNMT *(17q21-q22), *CEACAM5 *(19q13.1-q13.2), *PTPRA *(20p13), and *MMP9 *(20q11.2-q13.1). The advantage of the TRAC assay is that the expression levels of multiple genes can be measured simultaneously from a single sample thus lowering the amount of sample RNA required for the analysis. This is especially important for the analysis of often scarce clinical tissue samples.

TRAC analysis was performed at PlexPress (Helsinki, Finland). Custom TRACPackTM reagents for mRNA (PlexPress) were used in the analysis. Briefly, 90 μl of Hybridization Mix (containing labeled gene-specific detection probes and biotinylated oligo-dT probes) per well was dispensed to a 96-well PCR plate. Two micrograms of RNA sample was applied to each well in a 100 μl total reaction volume. An equal amount (30 amol/reaction) of single stranded 62-mer synthetic oligonucleotide hybridization control, including a poly-A tail, was added to each sample prior to hybridization. Hybridization was performed at 60°C, 650 rpm for 120 minutes (Thermomixer Comfort, Eppendorf, Hamburg, Germany). After hybridization affinity capture, purification, and elution were done using the KingFisher Flex (Thermo Fisher Scientific, Vantaa, Finland) magnetic particle processor. Streptavidin-coupled magnetic TRACPACK™ beads (50 μg, PlexPress) were added to the hybridization mixture and allowed to bind to the biotinylated mRNA-probe-oligo(dT)-hybrids for 30 minutes, after which the beads were washed 5 times using wash buffer to remove any unbound material. Labeled RNA-specific probes were eluted with elution buffer and detected by capillary electrophoresis, using the ABI3100 sequencer (Applied Biosystems, Cheshire, UK). The data was analyzed using the TRACParser software (PlexPress).

### Statistical analysis of the qRT-PCR data

A nonparametric Mann-Whitney test for two independent samples was applied to determine the statistical significance of differences in the relative mRNA expression levels of *ALPK2 *and *HHIPL2 *in nonmalignant and cancerous gastric samples as well as in gastric cancer samples of different histological subtypes or TNM-stages. A p-value < 0.05 was considered statistically significant (SPSS 17.0). In addition, in two separate analyses for gains and losses, the expression levels in cancer samples with copy number gains or losses (g1) were compared to cancer samples with normal copy number (g0) to assess the association between copy number and gene expression. Copy number data were available for 37 of the gastric samples included in the qRT-PCR analysis (Additional file 1: Clinical parameters). Gene expression fold changes were calculated by dividing the mean expression of one group (e.g. cancer samples) by the mean expression of the other group (e.g. nonmalignant samples).

### Statistical analysis of the TRAC assay data

A synthetic hybridization control was used in the data normalization to remove any non-biological variation in the data. For each target, signal intensities relative to this internal hybridization control were calculated. For the nine tissue samples analyzed in replicate mean signal intensity was used. A nonparametric Mann-Whitney test for two independent samples was applied to determine the statistical significance of differences in the relative mRNA expression levels of *ASAP1*, *CEACAM5*, *CYP3A4*, *ENAH*, *ERBB2, LTB4R*, *MMP9, OSMR*, *PERLD1*, *PNMT*, and *PTPRA *in nonmalignant and cancerous gastric samples as well as in gastric cancer samples of different histological subtypes or TNM-stages. A p-value < 0.05 was considered statistically significant (SPSS 17.0). The comparison of gene expression levels in samples with and without copy number alterations was performed as was described before for the qRT-PCR analysis. Copy number data were available for 43 gastric cancer samples included in the TRAC assay analysis.

## Results

### Gene copy number aberrations

All gene copy number changes in individual samples are shown in the Additional file 2: Copy number changes detected by aCGH analysis. Minimal common regions of recurrent (≥25%) alterations as well as their size, frequency, possible target genes, and chromosomal position in base pairs are shown in Table 2. The recurrent gained regions were located at 1q41-q43.1 (25%), 5p13.3-q11.1 (25%), 7q21.3-q22.1 (35%), 8q24.13-q24.3 (25%), 8q24.3 (45%), 14q11.2 (25%), 17q12-q21.1 (30%), 17q22-q24.2 (25%), 19q12-qter (35%), and 20p13-qter (40%). The recurrent deleted regions were located at 9p24.3-p21.1 (25%), 18q12.3-q22.2 (40%), 18q22.3-qter (35%), 21q11.2-q21.1 (30%), and Xq28 (25%). All recurrent copy number changes were detectable both in primary gastric cancers and in gastric cancer cell lines, except for the 14q11.2, which was altered only in five cell lines.

### Copy number associated gene expression changes

Altogether 256 individual genes (10% of all genes located in the recurrent chromosomal regions with copy number alterations) showed at least a 2-fold copy number associated change in their expression (range 2.0 - 34.6, median 3.8) (Additional file 3: Copy number associated gene expression changes). 226 of these genes were overexpressed and located in recurrent regions of copy number gains, whereas 30 genes were underexpressed and located in recurrent regions of copy number losses. Fold change in gene expression was calculated by comparing the expression levels of samples with copy number alterations to samples with normal copy number in a given gene. Therefore, a positive fold change refers to a copy number gain related increase in gene expression whereas a negative fold change refers to a copy number loss related decrease in gene expression.

*HHIPL2 *(HHIP-like 2) gene, amplified in the 1q41-q43.1 region, showed the highest copy number gain associated overexpression in gastric cancer according to the integrated microarray analysis (FC = 26.9). Generally, the highest gene expression fold changes between cancer samples with and without copy number gains were detected at the 19q region since out of the 40 genes showing >5-fold copy number associated changes in their expression, 19 (47.5%) were located in the 19q region (Additional file 3: Copy number associated gene expression changes). The most underexpressed gene in the recurrent regions of copy number losses was *ALPK2 *(alpha-kinase 2) (FC = -34.6) located at 18q12.3-q22.2.

Previously, three studies by us and others have been published that systematically integrate genome-wide copy number and gene expression data to identify genes whose expression has changed due to a copy number alteration in gastric cancer [15-17]. The comparison of the overlapping genes between these studies and the current study revealed 20 genes *TOMM20 *(1q42.3), *GGPS1 *(1q43), *CYP3A4 *(7q21.1), *MTAP *(9q21.3), *ASAP1 *(8q24.1-q24.2), *PPP1R1B *(17q12), *ERBB2 *(17q12-q21), *SERPINB3 *(18q21.3), *SERPINB8 *(18q21.3),*WDR7 *(18q21.2-q22), *HIF3A *(19q13.32), *ZNF480 *(19q13.33), *IL4I1 *(19q13.3-q13.4), *CST3 *(20p11.21), *PTPRA *(20p13), *SLC13A3 *(20q12-q13.1), *DDX27 *(20q13.13), *PARD6B *(20q13.13), *SGK2 *(20q13.2), and *TUBB1 *(20q13.32) that were either gained and overexpressed or deleted and underexpressed in our study and in at least one of the previously published studies. Previously published data together with the current results provide further evidence of the biological role of these genes in gastric cancer.

### Validation of potential gastric cancer target genes

Real-time qRT-PCR analysis showed that the expression of *HHIPL2 *was 7.4-fold higher in gastric cancer samples compared with the nonmalignant gastric tissues (p < 0.05). In addition, the overexpression of *HHILP2 *was significantly associated with copy number gain (p < 0.05) as the expression of *HHIPL2 *was 17.4-fold higher in cancer samples with copy number gain of *HHIPL2 *(g1) than in cancer samples with normal copy number of this gene (g0) (Tables 3 and 4). According to the qRT-PCR analysis there was a 2.9-fold underexpression of *ALPK2 *in gastric cancers with copy number losses (g1) compared with gastric cancers with normal copy number of *ALPK2 *(g0) (p < 0.05). Surprisingly, however, the expression of *ALPK2 *in gastric cancers in general was 1.9-fold higher (p < 0.05) than in the nonmalignant gastric tissues (Tables 3 and 4). Histological subtype or TNM-stage did not have a statistically significant effect on the expression of *HHIPL2 *or *ALPK2 *(Table 3).

**Table 3:** Results of the nonparametric Mann-Whitney test for the qRT-PCR and TRAC analysis data (SPSS17.0).

Gene	Chromosome	Cancer vs. non-malignant	intestinal vs. diffuse	g1 vs. g0	M0 vs. M1	T1-2 vs. T3-4	N0 vs. N1-3
*ALPK2*	18q21.31-q21.32	p < 0.05	*p *= 0.104	*p *< 0.05	*p *= 0.451	*p *= 0.072	*p *= 0.378
*ASAP1*	8q24.1-q24.2	p < 0.001	*p *= 0.319	*p *= 0.396	*p *= 0.208	*p *= 0.232	*p *= 0.289
*CEACAM5*	19q13.1-q13.2	p < 0.001	*p *= 0.061	*p *= 0.254	*p *= 0.543	*p *= 0.197	*p *= 0.253
*CYP3A4*	7q21.1	p < 0.001	*p *= 0.061	*p *< 0.05	*p *= 0.355	*p *= 0.228	*p *= 0.422
*ERBB2*	17q21.1	p < 0.001	*p *= 0.168	*p *< 0.05	*p *= 0.490	*p *= 0.350	*p *= 0.314
*HHIPL2*	1q41	p < 0.05	*p *= 0.248	*p < 0.05*	*p *= 0.847	*p *= 0.129	*p *= 0.736
*PNMT*	17q21-q22	p < 0.001	*p *= 0.649	*p *= 0.346	*p *= 0.133	*p *= 0.824	*p *= 0.136
*PERLD1*	17q12	p < 0.001	*p *= 0.316	*p *< 0.05	*p *= 0.437	*p *= 0.208	*p *= 0.161
*PTPRA*	20p13	p < 0.001	*p *= 0.304	*p *< 0.05	*p *= 0.112	*p *= 0.953	*p *= 0.596
*ENAH*	1q42.12	p < 0.001	*p *= 0.290	*p *< 0.05	*p *= 0.149	*p *= 0.949	*p *= 0.342
*LTB4R*	14q11.2-q12	p < 0.001	*p *= 0.427	*p *= 0.422	*p *= 0.468	*p *= 0.452	*p *= 0.604
*MMP9*	20q11.2-q13.1	p < 0.001	*p *= 0.495	*p *< 0.05	*p *= 0.089	*p *= 0.496	*p *= 0.238
*OSMR*	5p13.1	p < 0.001	*p *= 0.548	*p *< 0.05	*p *= 0.182	*p *= 1.000	*p *= 0.184

g1, gastric cancer samples with copy number gain/loss; g0, gastric cancer samples with normal copy number.

Multiplex gene expression analysis of 11 additional genes showing copy number gain associated overexpression according to the microarray analysis was carried out using the TRAC assay. All of these genes showed statistically significant differences in their mRNA expression in nonmalignant vs. cancerous gastric tissues. The p-values for each individual gene are shown in Table 3. The copy number gain related overexpression was detected for seven of these genes, including *CYP3A4*, *ENAH, ERBB2, MMP9, PERLD1, PTPRA*, and *OSMR *(p < 0.05, Table 3), which thereby validates the results from the integrated microarray analysis. Histological subtype or TNM-stage did not have a statistically significant effect on the expression of the tested genes (Table 3).

## Discussion

Gene copy number alteration is known to be an important mechanism for a cancer cell to regulate the expression of cellular proto-oncogenes and tumor suppressor genes. Recent studies by us and others have demonstrated that 10-15% of all gene expression changes are directly associated with gene copy number changes and 10-45% of the amplified genes are overexpressed in different epithelial tumors and cell lines [16,17,21,22]. In the present study, our aim was to screen for those genes that are differentially expressed in association with copy number alteration and to identify potential molecular markers with a biological role in gastric carcinogenesis. Our approach was to screen for DNA copy number changes using a high-resolution array-based analysis combined with measurement of transcriptional activities of the genes located in the recurrent regions of copy number alterations using both gene expression arrays as well as qRT-PCR and TRAC analyses. On the whole, we identified recurrent copy number gains in 10 chromosomal regions and losses in 5 regions, which are in concordance with the previous studies [8-17,29]. The majority of the identified gains and losses were observed in multiple tumors and cell lines suggesting genomic alterations with an important biological role in gastric cancer.

Altogether, 10% of all the genes located in the recurrent regions of copy number alterations were over- or underexpressed along with the copy number change. This is in line with previous reports on the impact of copy number on gene expression in solid tumors [17,18,20-22]. A literature search showed that 50 of these genes (37 up- and 13 down-regulated genes) had previously been reported to have mutations, polymorphisms, copy number and/or expression changes in malignant tumors, and 4 of the genes (*ERBB2*, *JAK2*, *LIFR*, and *ZNF331*) are included in the Cancer Gene Census of the Wellcome Trust Sanger Institute [30]. Furthermore, 14 of the identified genes (*AGT*, *APOC1*, *APOE*, *AXIN2*, *CEACAM5, ERBB2*, *HSPA13*, *ID1*, *KLK12*, *MMP9*, *PPP1R1B*, *PTPRA*, *SERPINB5*, and *SMAD7*) have previously been associated with malignant gastric tumors.

In the current study, the association between copy number and gene expression varied among different genes. *ALPK2 *showed the strongest association between copy number loss and underexpression according to the integrated microarray analysis. The frequency of copy number loss of *ALPK2 *in our data was 40%. The copy number associated underexpression in gastric cancers was validated with qRT-PCR analysis as *ALPK2 *showed a 2.9-fold underexpression in gastric cancers with copy number losses (g1) compared with gastric cancers with normal copy number of *ALPK2 *(g0). However, the underexpression of *ALPK2 *in gastric cancers in general compared to normal gastric tissues was not detected. *ALPK2 *is located in the 18q12.3-q22.2 region, a region of recurrent genomic loss in gastric cancers. No previous publications regarding the possible tumor association of *ALPK2 *or its function in normal tissues have been published. The 18q region is also known to harbor two well-known gastric cancer associated tumor suppressor genes *DCC *(18q21.3) and *SMAD4 *(18q21.1) [15,31,32]. However, these genes did not show a correlation between copy number and gene expression in our data.

The *HHIPL2 *gene showed the highest copy number gain associated overexpression according to the integrated microarray analysis. The frequency of copy number gain of *HHIPL2 *in our data was 25%. The overexpression was validated with the qRT-PCR analysis as *HHIPL2 *showed a 7.4-fold overexpression in gastric cancers compared to the normal tissues. Furthermore, the expression was 17.4-fold higher in gastric cancers with copy number gains compared to the gastric cancers with normal copy number of this gene. This is the first study to report an association of *HHIPL2 *with gastric cancer. HHIPL2 is a transmembrane protein containing a short N-terminal cytoplasmic region. It belongs to the HHIP gene family and is expressed in the testis, thyroid gland, osteoarthritic cartilage as well as in pancreatic and lung cancers [33]. Overexpression of *HHIPL2 *has not been previously associated with any cancers and its exact biological function is not known. However, another member of the HHIP family, HHIP1, is known to interact with proteins of the Hedgehog signaling pathway [33]. This association could possibly also offer an explanation for *HHIPL2*'s role in gastric cancer.

To further highlight the clinical significance of the genes mapping to recurrent copy number altered regions in gastric cancers as well as to validate the microarray results, eleven genes were selected for the affinity capture based transcript analysis (TRAC assay). These eleven genes were selected based on their copy number associated gene expression changes detected in the integrated microarray analysis, as well as based on their previously published associations with cancer. The TRAC assay has previously been shown to correlate well with the conventional qRT-PCR and Northern blot analyses [28,34]. The TRAC analysis validated the results obtained from the microarray analysis since all of the genes showing overexpression in the microarray analysis also showed an increased expression in the TRAC analysis. Seven out of these genes also showed copy number gain associated overexpression. Overexpression in samples with copy number alterations compared with samples with normal copy number ranged from 1.7 to 3.8-fold (Table 4) and in gastric cancers in general these seven genes were overexpressed 3.5 to 8.9-fold compared with normal tissues (Table 4).

**Table 4:** Genes showing an association between copy number and expression in gastric cancer.

Gene	Chromosome	Fold change cancer vs. normal	*p*-value cancer vs. normal	Fold change g1 vs. g0	*p*-value g1 vs. g0	Previous reports in gastric cancer	PubMed IDs
*ALPK2*	18q21.31-q21.32	1.9	p < 0.05	-2.9	p < 0.05	-	-
*CYP3A4*	7q21.1	8.9	p < 0.001	2.4	p < 0.05	polymorphisms	17605821
*ENAH*	1q42.12	8.4	p < 0.001	3.8	p < 0.05	-	-
*ERBB2*	17q21.1	3.5	p < 0.001	1.8	p < 0.05	amplification and overexpression	14991576, 19156142, 17555797
*HHIPL2*	1q41	7.4	p < 0.05	17.4	p < 0.05	-	
*MMP9*	20q11.2-q13.1	4.8	p < 0.001	1.7	p < 0.05	overexpression, polymorphisms	18437914, 18451255, 16237750
*OSMR*	5p13.1	3.4	p < 0.001	2.4	p < 0.05	-	-
*PERLD1*	17q12	3.4	p < 0.001	3.0	p < 0.05	amplification and overexpression	16849520
*PTPRA*	20p13	4.1	p < 0.001	1.3	p < 0.05	overexpression	16338072

Gene expression fold changes according to the qRT-PCR and TRAC analyses and *p*-values from nonparametrical Mann-Whitney test are shown (SPSS 17.0). g1, gastric cancer samples with copy number gain/loss; g0, gastric cancer samples with normal copy number.

*ERBB2 *and *PERLD1 *have been previously been reported to be gained and overexpressed in gastric cancers [17,23,35]. Both of these genes are part of the PPP1R1B-STARD3-TCAP-PNMT-PERLD1-ERBB2-MGC14832-GRB7 locus at the 17q12 region, which has previously been reported to be gained and overexpressed in breast and gastric cancers [23,35-38]. We have previously reported *PERLD1 *to have a copy number gain in 18.4% of primary gastric tumors [17]. In the current study, *PERLD1 *was amplified in 30% gastric cancers and copy number gain caused a 3.0-fold overexpression (p < 0.05) of this gene.

In addition to *ERBB2 *and *PERLD1*, the TRAC analysis also identified five novel genes, *CYP3A4*, *ENAH*, *MMP9*, *PTPRA*, and *OSMR*, which have not been reported as gained and overexpressed in gastric cancer before. Of these *OSMR *and *ENAH *are especially interesting since they have no previous link to gastric carcinogenesis. Oncostatin M (*OSM*) is a member of the interleukin-6 cytokine family that binds to its receptor, *OSMR*, to induce signals important to hematopoiesis, inflammation, bone remodelling, heart development, and neurogenesis [39]. *ENAH *is an actin binding protein involved in the regulation of cell motility [40]. The frequency of copy number gain for both *OSMR *and *ENAH *in our data was 25%. *OSMR *and *ENAH *showed overexpression in samples with copy number gains compared to samples with normal copy number (p < 0.05, FC 2.4 for *OSMR *and 3.8 for *ENAH*) as well as in gastric cancers in general compared to the normal gastric tissues (p < 0.001, FC 3.4 for *OSMR *and 8.4 for *ENAH*). Ng *et al. *(2007) [41] demonstrated that gene copy number and expression levels of *OSMR *were increased in cervical squamous cell carcinomas and that overexpression was associated with poor survival of these patients. However, to our knowledge no previous studies exist that link *OSMR *expression and copy number changes to gastric carcinogenesis. The overexpression of *ENAH *has previously only been reported in breast cancer [42].

The gastric cancer-related overexpression of *PTPRA *and *MMP9 *has previously been implicated [43-45] but the association between copy number and overexpression has not been reported. The role of *PTPRA *in gastric cancer might be linked to its biological role in integrin signalling, cell adhesion, and activating the SRC family tyrosine kinases. *MMP9 *is known to be overexpressed many epithelial tumors including gastric tumors [46-48] and its involvement in the breakdown of extracellular matrix could explain its role in gastric cancer progression and formation of metastases. The overexpression of *CYP3A4 *has been reported in breast cancer [49] but not in gastric cancer. In all, the combined microarray and transcript analysis highlighted several interesting genes as potential target genes for gastric cancer.

## Conclusions

To conclude, the present results prove that integrated analysis of gene copy number and expression levels is an effective approach in identifying potential biomarkers for gastric cancer. All of the genes, identified based on their association of copy number and gene expression in the microarray analysis, were also differentially expressed in cancerous gastric samples compared to nonmalignant tissues according to the qRT-PCR and TRAC analyses. Copy number-associated gene expression changes were confirmed for 9 out of the 13 (69.2%) genes (*ALPK2*, *CYP3A4*, *ENAH*, *ERBB2*, *HHIPL2*, *MMP9*, *PERLD1*, *PTPRA*, and *OSMR*) thereby validating the results from the integrated microarray analysis and highlighting these genes as potential biomarkers for gastric cancer. Further studies are however required to decipher their biological significance in gastric cancer initiation and progression.

## Competing interests

The authors declare that they have no competing interests.

## Authors' contributions

SJ carried out the nucleic acid extractions, microarray hybridizations, microarray data analysis, qRT-PCR, and TRAC-assay analyses and participated in study design and cell culturing and drafted the manuscript. PP and AK collected all patient material and participated in study design, M-L. K-L. performed the histological analysis of the gastric tissue samples. OM supervised the study. All authors read and approved the final manuscript.

## Pre-publication history

The pre-publication history for this paper can be accessed here:

http://www.biomedcentral.com/1471-2407/10/73/prepub

## Supplementary Material

Additional file 1 Clinical parameters. Click here for file

Additional file 2 Copy number changes in gastric tumors and cell lines detected by aCGH. Click here for file

Additional file 3 Copy number associated gene expression changes. Click here for file
