# Supplementary material for: Genome-wide gene copy number and expression analysis of primary gastric tumors and gastric cancer cell lines
Source: BMC Cancer. 2010 Mar 1;10:73. doi: 10.1186/1471-2407-10-73 (PMC2837868; doi:10.1186/1471-2407-10-73)
Supplement: Additional file 1 — Clinical parameters. [file 1471-2407-10-73-S1.PDF]

Additional file 1: Clinical parameters.

Gastric tissues	Location	Histology	Age/sex	T	N	M	CN data	TRAC assay	qRT-PCR
126A	Corpus	Non-malignant	56/F				no	yes	yes
128A	Corpus	Non-malignant	58/M				yes	yes	yes
134A	Corpus	Non-malignant	65/F				yes	yes	yes
142A	Antrum	Non-malignant	50/M				no	yes	yes
142C (repl. 142A)	Antrum	Non-malignant	50/M				no	yes	no
146B	Antrum	Non-malignant	50/F				no	yes	yes
153A	Antrum	Non-malignant	75/M				no	yes	yes
160B	Corpus	Non-malignant	67/M				no	yes	yes
163C	Antrum	Non-malignant	63/M				no	yes	yes
23B	Antrum	Non-malignant	67/F				yes	yes	yes
G22N	Antrum	Non-malignant	56/F				no	no	yes
G23N	Cardia	Non-malignant	41/F				no	no	yes
G24N	Corpus	Non-malignant	76/M				no	no	yes
G28N	Antrum	Non-malignant	57/F				no	no	yes
G30N	Antrum	Non-malignant	55/M				no	no	yes
G32N	Antrum	Non-malignant	68/M				no	no	yes
G33N	Cardia	Non-malignant	59/M				no	no	yes
G37N	Antrum	Non-malignant	58/M				no	no	yes
T105A	Antrum	Non-malignant	65/F				yes	yes	yes
T10B	Corpus	Non-malignant	83/M				no	yes	yes
T10C (repl. T10B)	Corpus	Non-malignant	83/M				no	yes	no
T114A	Corpus	Non-malignant	67/M				no	yes	yes
T125A	Antrum	Non-malignant	66/M				no	yes	yes
T128A	Antrum	Non-malignant	72/F				no	yes	yes
T131A	Corpus	Non-malignant	75/F				no	yes	yes
T140B	Antrum	Non-malignant	64/M				no	yes	yes
T144A	Antrum	Non-malignant	83/F				no	yes	yes
T171A	Antrum	Non-malignant	81/F				no	yes	no
T172A	Antrum	Non-malignant	68/M				no	yes	yes
T20A	Corpus	Non-malignant	58/M				yes	yes	yes
T25A	Antrum	Non-malignant	70/M				no	yes	yes
T27A	Corpus	Non-malignant	58/M				no	yes	yes
T40A	Antrum	Non-malignant	53/M				yes	yes	yes
T44A	Antrum	Non-malignant	75/M				yes	yes	yes
T45A	Antrum	Non-malignant	64/M				no	yes	no
T48A	Corpus	Non-malignant	65/M				no	yes	no
T49A	Antrum	Non-malignant	42/F				no	yes	no
T54A	Corpus	Non-malignant	63/F				no	yes	yes
T80A	Corpus	Non-malignant	33/M				no	yes	yes
T80B	Corpus	Non-malignant	33/M				no	yes	no
T88A	Corpus	Non-malignant	81/M				no	yes	yes
T92A	Antrum	Non-malignant	72/M				no	yes	yes
T93B	Corpus	Non-malignant	77/M				no	yes	yes
T34A (repl.T34C)	Corpus	Intestinal	75/M	2	0	0	no	yes	no
125A	Antrum	Intestinal	80/M	3	1	1	yes	yes	yes
141A	Corpus	Intestinal	72/M	3	3	0	no	yes	yes
144B	Antrum	Intestinal	79/F	n/a	n/a	n/a	yes	yes	yes
154A	Antrum	Intestinal	75/M	1	0	0	yes	yes	yes
162A	Antrum	Intestinal	67/M	3	0	0	no	yes	yes
162B (repl. 162A)	Antrum	Intestinal	67/M	3	0	0	no	yes	no
164A	Corpus	Intestinal	63/M	3	2	0	yes	yes	yes
200A	Corpus	Intestinal	57/F	n/a	n/a	n/a	yes	yes	yes
22A	Corpus	Intestinal	91/F	2	1	0	no	yes	no
3TC	Corpus	Intestinal	57/F	n/a	n/a	n/a	yes	yes	yes
41A	Antrum	Intestinal	83/F	n/a	n/a	n/a	no	yes	no
4T/N (tb)	Corpus	Intestinal	72/M	n/a	n/a	n/a	yes	yes	yes
54B	Antrum	Intestinal	72/F	3	2	1	yes	yes	yes
90A	Antrum	Intestinal	76/F	3	1	0	yes	yes	yes
96A	Antrum	Intestinal	64/M	2	1	0	yes	yes	yes
G32T	Antrum	Intestinal	68/M	3	1	0	no	no	yes
T102A	Antrum	Intestinal	82/M	2	1	0	no	yes	yes
T119B	Corpus	Intestinal	76/M	3	0	0	yes	yes	yes
T121A	Antrum	Intestinal	71/M	n/a	1	0	no	yes	yes
T12B	Corpus	Intestinal	87/F	3	1	0	yes	yes	yes
T136A (repl.54B)	Antrum	Intestinal	72/F	3	2	1	no	yes	no
T142A	Antrum	Intestinal	64/M	2	1	0	yes	yes	yes
T143A	Antrum	Intestinal	82/F	3	2	1	yes	yes	no
T148A	Antrum	Intestinal	83/F	2	1	0	yes	yes	yes
T161A	Corpus	Intestinal	91/F	2	1	0	yes	yes	yes
T16A (repl. 90A)	Antrum	Intestinal	76/F	3	1	0	no	yes	no
T177A	Corpus	Intestinal	75/M	4	2	0	yes	yes	yes
T181A	Antrum	Intestinal	61/M	2	2	0	no	yes	yes
T23A	Corpus	Intestinal	70/M	3	2	0	yes	yes	yes
T2A	Antrum	Intestinal	61/M	4	1	0	yes	no	no
T34C	Corpus	Intestinal	75/M	2	0	0	yes	yes	yes
T47B	Antrum	Intestinal	65/M	3	2	1	yes	yes	no
T51B	Corpus	Intestinal	69/M	4	1	0	yes	yes	yes
T51C (repl. T51B)	Corpus	Intestinal	69/M	4	1	0	no	yes	no
T5A	Antrum	Intestinal	99/M	3	1	0	yes	yes	no
T87A	Antrum	Intestinal	81/F	3	1	0	yes	yes	yes
T8A	Corpus	Intestinal	60/M	3	2	0	yes	yes	yes
T91A	Corpus	Intestinal	72/M	3	1	1	no	yes	no
T91B (repl.T91A)	Corpus	Intestinal	72/M	3	1	1	yes	yes	yes
T94A	Corpus	Intestinal	77/M	3	2	0	yes	yes	yes

T94B (repl.T94A)	Corpus	Intestinal	77/M	3	2	0	no	yes	no
135A	Antrum	Diffuse	65/F	2	0	0	yes	yes	yes
139A	Corpus	Diffuse	74/M	3	1	1	yes	yes	yes
148A	n/a	Diffuse	68/F	3	3	1	yes	no	no
151B	Corpus	Diffuse	50/F	n/a	n/a	n/a	yes	yes	yes
158A	angulus	Diffuse	40/F	3	2	0	no	yes	no
G22T	Antrum	Diffuse	56/F	2	2	0	no	no	yes
G24T	Corpus	Diffuse	76/M	2	1	0	no	no	yes
G28T	Antrum	Diffuse	57/F	2	1	0	no	no	yes
G31T	Corpus	Diffuse	61/F	2	0	0	no	no	yes
G33T	Cardia	Diffuse	59/M	2	2	0	no	no	yes
G51T	Corpus	Diffuse	79/F	2	0	0	no	no	yes
G54T	Cardia	Diffuse	81/M	2	2	0	no	no	yes
G55T	Antrum	Diffuse	56/F	3	2	0	no	no	yes
T104A	Corpus	Diffuse	62/F	3	2	0	yes	yes	no
T112A	Corpus	Diffuse	78/F	2	0	0	yes	yes	no
T139A	Antrum	Diffuse	64/M	3	1	0	yes	yes	yes
T14B	Antrum	Diffuse	80/F	3	2	1	yes	yes	yes
T156A	Corpus	Diffuse	81/F	3	2	0	yes	yes	no
T50A	Corpus	Diffuse	42/F	3	2	1	yes	no	no
T53A	Antrum	Diffuse	63/F	3	2	0	yes	yes	yes
T78A	Corpus	Diffuse	84/F	3	1	0	no	no	no
T79A	Antrum	Diffuse	33/M	4	0	0	no	yes	no
T84A	Antrum	Diffuse	53/F	3	1	1	yes	no	no
T95A	Antrum	Diffuse	38/F	3	3	1	no	yes	yes
T97A	Antrum	Diffuse	41/M	4	2	1	yes	yes	yes
G38T	Corpus	Cancer (histology n/a)	64/M	3	1	0	no	no	yes

**Cell lines**	**Source**	**Histology**	**Age/sex**	**T**	**N**	**M**	**CN data**	**TRAC assay**	**qRT-PCR**
AGS	Primary tumor	Adeno-carcinoma	54/F				yes	yes	yes
KATOIII	Pleural effusion	Diffuse	55/M				yes	yes	yes
MKN1	Lymph node metastasis	Adeno-squamous carcinoma	72/M				yes	yes	yes
MKN7	Lymph node metastasis	Intestinal	39/M				yes	yes	yes
MKN28	Lymph node metastasis	Intestinal	70/F				yes	yes	yes
MKN45	Liver metastasis	Diffuse	62/F				yes	yes	yes
TMK-1	Lymph node metastasis	Diffuse	21/M				yes	yes	yes

CN=copy number
repl.=replicate of
