# Supplementary material for: Genome-wide gene copy number and expression analysis of primary gastric tumors and gastric cancer cell lines
Source: BMC Cancer. 2010 Mar 1;10:73. doi: 10.1186/1471-2407-10-73 (PMC2837868; doi:10.1186/1471-2407-10-73)
Supplement: Additional file 2 — Copy number changes in gastric tumors and cell lines detected by aCGH. [file 1471-2407-10-73-S2.PDF]

Additional file 2. Copy number changes in gastric tumors and cell lines detected by aCGH.

Sample	Copy number gains	Copy number losses
14TA	7p22.1-p11.2, 7p11.2, 7q11.23-q21.3, 8p22-p21.3, 9p24.3-q21.13, 18p11.32-p11.31, 18p11.21-q11.2	2q35, 2q37.1-q37.3, 3p14.3, 4p16.3-p16.1, 5p15.33-p15.31, 5q32, 6p24.3, 6p21.32, 6q27, 7q34, 7q36.1-q36.3, 8p11.21-q24.22, 9q33.3-q34.3, 10p15.3-p15.1, 10q11.22-q11.23, 10q22.1, 10q22.3, 10q26.2-q26.3, 11p15.5-p15.4, 11q25, 12q24.22, 14q32.12,-q32.33, 14q32.33, 16q23.2, 16q24.1-q24.3, 17p13.3-p13.1, 17q21.33, 17q25.1-q25.3, 18q21.1, 20q13.33, 21q22.2-q22.3, 22q11-q13.33, Xp11.23
200A	1p13.3-q44, 1p11.2-q23.1, 1q32.1, 1q44, 2p25.3-q37.3, 2p25.3-p21, 2p23.3-p23.2, 2p16.1-p12, 2p11.2-q14.3, 2q13-q14.1, 2q33.1-q33.2, 2q35-q37.2, 7p22.3-p22.1, 7p11.2-q11.23, 7q21.3-q22.1, 7q32.1-q32.2, 7q34, 8p11.23-p11.22, 8p11.21-q24.3, 8q11.21-q24.3, 8q11-q22.1, 8q24.13-q24.21, 8q24.3, 12q13.3-q14.1, 13q12.11-q34, 13q12.11-q12.3, 13q14.11-q14.3, 13q21.33-q13.1, 13q32.2-q32.3, 15q11.2, 16p13.3-q24.23, 16p13.3.-p11.2, 16q11.2-q24.3, 16q21-q22.1, 16q22.1, 17q11.1-q25.3, 17q11.2-q12, 17q21.32-q21.33, 17q21.33-q24.3, 19q12-q13.43, 20p12-q13.33, 20p13, 20p12.1, 20p11.1-q11.1, 20q11.21-q13.2	1p36.32-p21.1, 1p32.3-p21.1, 1q21.3, 2q24.2, 2q37.2, 3p25.3-q29, 3p14.2, 3p12.3-q12.1, 3q12.3-q13.32, 3q21.1-q21.3, 3q24, 3q26.1-q26.2, 3q26.31-q26.32, 4p16.3-q35.2, 4p16.1, 4p14-p13, 4q12-q13.2, 4q28.2-q28.3, 4q35.1-q35.2, 5p15.1-p13.3, 5q11.2, 5q14.2-q23.3, 5q23.2, 7q34, 8p23.2, 9p24.3-q34.12, 9p23, 9p23-p21.1, 9q33.3, 9q34.11-q34.12, 10q11.22, 10q11.23-q21.3, 10q22.3-q23.1, 11q22.3, 12q21.1-q21.33, 14q12-q21.3, 14q24.3-q31.3, 14q32.33, 16p11.2, 17p13.1-p12, 18p11.32-q23, 18q21.31, 20p13, 21p11.1-q22.3, 21q21.1-q21.3, Xp22.33-q28, Xp21.3-p21.1, Xp11.3-p11.23, Xq12-q13.1, Xq21.1-q22.1, Xq26.3-q28, Xq28
222A	1q21.3, 3q27.1-q27.3, 7p22.3-p22.1, 7p11.2-q22.3, 7q21.3-q22.3, 8q24, 9p13.3-p13.2, 10q11.21, 10q22.2, 10q25.2-q25.3, 10q26.13, 16p13.3-p11.2, 17q12-q21.2, 17q12-q21.1, 17q21.2, 20p11.21-p11.23	3p24.2-p24.1, 3p14.2, 3q26.1, 4q13.2, 6p21.32, 9p24.3-p21.1, 9p21.3-p21.1, 11q11, 13q21.21-q31.1, 21q11.2-q22.1
232A	8p23.2-q24.1, 13q11-q34, 19p12-q13.43, 20p13-q13.33	2q37.1-q37.3, 5p15.33-p15.2, 11p15.4, 11q11, 11q24.2-q25, 14q32.13, 18q12.2-q23, Xp22.31, Xq24-q25, Xq27.2-q28
3TC	1q44, 2p25.3-q37.3, 2p25.2-p21, 2p24.3-p24.1, 2p12-q14.3, 8p11.23-p11.22, 11p15.2-p14.3, 11q12.2-q22.1, 11q12.2-q13.3, 12q13.3-q14.1, 15q11.2, 16p11.2-q24.3, 17q11.1-q25.3, 17q21.32-q21.33, 19q12-q13.43, 20p13-q13.33, 20p12.1	1p36.33-p21.1, 1q21.3, 3p25.3-q29, 3p14.2, 4p16.3-q35.2, 4q22.1, 5q12.1, 5q15-q22.2, 7q34, 9p24.3-q34.3, 9p23, 9q34.11-q34.12, 10q11.22, 14q32.33, 16p11.2, 16q22.1, 18q21.31, 20p13, 21q11.2-q22.3, Xp22.33-q28

4T/N	7q22.1, 22q11.23, Xp22.33-q28, Xp21.1-q22.1	3p14.2, 6p21.32, 7q31.1, 8p11.23-p11.22, 9p24.1-p23, 13q11-q34, 14q11.2, 15q11.2, 16q23.1, 22q11.23
10TB	No changes	No changes
17TA	7q11.21-q36.3, 8p23.3-q24.3	4q13.2, 7q11.21, 11q11, 14q11.2, 22q11.23
185B	8p11.23-p11.22, 8q24.3, 11p15.5-p15.4	No changes
1AT/N	Xp21.1-p11.23, Xq28	8p11.23-p11.22, 12p13.2, 14q32.33
6TB	7p22.3-p22.1, 7q11.21-q11.23, 7q21.3-q22.1, 8p11.23-p11.22, 8q24, 12q15, 14q11-q11.2, 14q32.33, 16p13.3, 20p13-q13.33	1q21.3, 4q13.2, 6p25.3, Xq21.1-q22.1, Xq24-q25
9TD	1p11.2-q44, 5p14.1, 5p13.3-q11.1, 5p13.1-q11.1, 6q22.33-q25.1, 8p11.23-p11.22, 8q22.1-q24.3, 11p15.5-p15.4, 11q13.2-q13.4, 19p13.2-q13.43, 20p13-q13.33	1q21.3, 2q14.3-q24.3, 3p14.2, 3q26.1, 4q13.2, 6p21.32, 6q26, 14q32.33, 16q12.2, 22q11.23, 22q13.1, Xp22.33-q28, Xp22.31-q11.2
13TA	8p11.23-p11.22, 18q11.2	14q32.33
AGS	1q12-q44, 1q21.2-q23.1, 1q31.1-q31.3, 6p25.3-p22.3, 6q27, 8p11.23-p11.22, 14q11.2-q32.33, 14q12, 14q21.1-q21.3, 16p11.2, 18p11.32, 19q12-q13.43, 20p13-q13.33	1q25.1, 2p12-p11.2, 2q22.1-q22.3, 3p23, 3p14.2, 3q26.31, 3q29, 4q22.1, 6p21.32, 7p21.3, 7p14.1, 7q31.1, 7q35, 8p23.3-p23.2, 8p23.2, 8q21.11, 9p24.1-p23, 10q21.1, 10q22.2, 14q23.3, 16q22.1, 16q23.1, 18q11.2-q23, 22q13.1, Xp22.31
KatoIII	2p25.1, 3p22.1, 3q21.2-q29, 3q21.3, 6p22.1-p21.1, 7p22.3-q36.3, 7p22.3-q21.11, 7p22.3, 7q11.23-q21.11, 7q21.11-q35, 7q21.3-q22.1, 8q24.3, 9q33.2-q34.3, 10q26.12, 10q26.12-q26.13, 11p11.2, 11q12.2-q25, 11q12.2-q13.2, 11q14.1-q21, 11q22.3-q25, 16p13.3, 16q24.2-q24.3, 17p13.3-p13.1, 17p12-q25.3, 19q13.11-q13.43, 20p12.3-p12.1, 20p12.1-q13.33, 22q11.1-q13.33, Xp22.33-q28, Xp21.1-q13.1	1p33-q44, 1q42.13-q44, 2p12, 2q11.1-q35, 2q11.2-q22.1, 2q21-q22.1, 2q23.2-q34, 2q35-q37.3, 2q37.3, 3p26.3-p12.2, 3p14.2, 3q13.2, 4p16.3-q35.2, 4p13.2, 5p15.33-q35.1, 5p15.2, 6q25.1, 7q31.1, 10p15.3-q26.11, 10q26.2-q26.3, 11p11.12, 12q21.2-q21.32, 15q11.2-q12, 17p13.1, 18p11.32-q23, 19p13.3-q12, 20p12.1, 21q11.2-q22.3, 21q21.1-q21.3, 21q22.3, 22q11.23, Xp22.31
MKN-1	1q41-q44, 2p24.1-p23.3, 2q24.3, 5p15.33-q11.1, 6p25.1-p24.3, 7q11.23, 7q21.12, 8p11.23-p11.22, 8q24.13-q24.3, 9p22.3-22.2, 9p21.1-q34.3, 9p21.1, 9q31.3-q32, 10q22.2, 11p15.55-p11.12, 11p15.3, 11q13.2, 11q14.1, 11q22.2, 12p12.1-p11.21, 12p1-p11.23, 12p11.23-p11.22,	1q31.2, 2p23.3-p23.2, 2p16.3, 2q33.3, 3p26.3-p11, 3p14.2, 3q26.1, 3q26.31, 4p12-q13.1, 4q13.2, 4q21.21, 5q31.2-q31.3, 6p25.3-p25.1, 6p21.32-p21.2, 6q26, 7q22.1, 8p23.3-q11.21, 9p24.3-p21.2, 9p21.2-p21.1, 10p15.3-q26.3, 10p12.2-p12.1, 10q23.33, 10q26.11, 11q11,

	12q13.33, 12q24.12-q24.13, 14q24.3-q32.33, 15q14, 16p12.3, 17q11.1-q11.2, 17q21.1-q23.2, 17q21.2-q21.31, 19p13.2-q13.2, 19p13.2-p12, 19q12-q13.12, 19q13.33-q13.41, 20p13-p12.2, 20q11.21-q13.33, 20q11.23-q12, 20q12-q13.12, 20q13.12-q13.2, 21q22.11, Xp22.33-q28, Xq22.1	11q12.2-q13.1, 12p13.33-p12.1, 14q11-q24.3, 14q11.2, 15q11.2-q26.3, 15q23, 16p13.3-q24.3, 15q23, 16p13.3-q24.3, 16p13.3-p12.3, 16q11.2-q24.3, 17q11.2-q12, 18p11.32-p11.22, 19p13.3-p13.2, 19q13.2-q13.43, 21p11.2-p11.1, 22q11.1-q13.33, 22q13.1, Xp11.4, Xq21.33
MKN-7	1p32.33, 1p31.3-p31.1, 1q21.2-q23.3, 1q22, 1q42.3, 2p25.1-p12, 2p16.2-p16.1, 2q23.3-q32.1, 3p21.31, 5p15.33-q11.1, 5q13.2-q14.1, 5q32-q33.1, 6q13, 7p22.2-q11.21, 7p13-q11.21, 8p12-q11.21, 8q21.2-q24.12, 8q24.13-q24.22, 8q24.13-q24.21, 9p21.1-p12, 9p22.31-q22.33, 9q31.3-q32, 9q33.2-q34.2, 11p15.5-p15.4, 11p15.4-p15.3, 11q11.1-q24.3, 14q11.2-q21.3, 14q21.3, 15q15.1, 15q21.3, 15q26.1, 16p13.12-p11.2, 17p13.3-p11.2, 17q12-q21.1, 17q21.2, 17q21.31, 17q22-q24.2, 18p11.32-q11.2, 19p13.3-q13.43, 19q12, 19q13.3-q13.43, 19q13.11-q13.12, 19q13.11, 20p13-q13.33, 20p12.3-p11.21, 20q11.21-q13.31, 22q11.21-q11.23, 22q13.2, Xp22.31-q27.2, Xp21.2-p11.4	1p36.33-p36.31, 1p33-p32.3, 1p31.1-p11.2, 1q44, 2p22.1, 2p12-p11.2, 2q12.1-q23.3, 2q21.3, 2q22.3, 3p14.2, 3q26.1, 3q26.31, 4p16.3-q22.1, 4p14-p13, 4q22.1, 4q35.1, 6p21.33, 6q11.1-q12, 6q14.1-q23.2, 6q27, 7q31.1-q32.1, 7q32.2-q33, 8p23.3-p12, 8q22.1, 9p24.3-p21.1, 9p22.1-p21.3, 9q21.11-q22.2, 10p15.3-q26.3, 10q11.22-q21.2, 10q26.2-q26.3, 11q11, 11q11-q12.1, 12p13.33-q21.32, 12p13.2, 12q21.31, 13q14.3-q34, 15q11.2-q22.31, 18q22.2-q23, 19q12-q13.11, 20p12.1, 22q12.3-q13.33, Xp22.31
MKN-28	1p36.33-p36.22, 1p36.33-p36.32, 1p36.32-p36.23, 1p36.22, 1p22.3-p12, 1p22.3-p21.2, 1p21.2-p12, 2p15, 3p26.3, 3p12.1-q12.1, 3p12.1-p11.1, 3p11.1-q12.1, 3q25.1, 6p21.32, 7p22.2-p15.3, 7p22.3-p21.3, 7p21.1-p15.3, 8q21.2-q24.2, 8q21.2-q23.3, 8q22.2, 8q24.12-q24.23, 9p21.1-q34.3, 9p13.3-p13.2, 9q13-q33.2, 9q33.3-q34.3, 11q11-q25, 11q12.2-q13.5, 11q14.1-q22.3, 12q14.3, 12q15, 12q24.31-q24.33, 13q13.3, 14q11.2, 15q11.2-q26.3, 15q26.2, 16p13.3, 19q13.43, 20p13-q13.33, 20p12.3-p12.1, 22q11.1-q11.21, 22q11.21, Xq21.33-q22.3, Xq23	1p36.33, 1q21.3, 3p22.1-p14.1, 3p21.31-p21.1, 3p14.2, 3q13.2-q13.31, 3q26.1, 5q23.1, 7q11.21-q21.11, 7q11.23, 7q21.11, 8p23.3-p23.2, 8p21.3, 8p11.23-p11.22, 9p24.3-p21.1, 9p21.2, 10p12.31-p12.1, 10q11.21-q21.1, 11q11, 12p11.23-p11.22, 12q13.2-q13.3, 14q31.3-q32.33, 14q32.33, 16p13.3, 16q23.1, 16q24.2-q24.3, 18p11.32-q23, 18q12.1-q12.3, 18q22.1-q22.3, 19p13.3, 20q13.33, 21q11.2-q21.1, Xp22.33-q28, Xp22.33-q21.33, Xp21.3-p21.1, Xp11.3-p11.23, Xq21.2-q21.33, Xq23-q28, Xq28
MKN-45	1p13.3, 4p15.31, 4p15.2, 4p13-p12, 4q24, 5p15.33-q35.2, 5p15.33-p13.2, 5p13.3-q35.2, 6p25.3-q12, 6q24.1-q24.3, 7p21.3-q11.22, 7q11.23-q31.1, 7q31.1-q31.32, 7q36.1-q36.3, 8q21.3-q24.3, 9q34.2-q34.3, 10p14-q26.3, 11p14.3-q22.1, 11p14.2-p14.1, 11q13.5-q14.1, 11q14.2-q21, 11q21-q22.1, 12p13.2-q21.2, 12p12.1-p11.1, 12p11.1-q14.2, 12q14.3-q21.1, 13q31.3-q34, 14q11.2-q32.33, 15q22.2-q26.3, 18p11.32-q12.3, 20p13-p12.3, 20p12.2-p12.1, 20p11.22-q13.33, 21q11.2-q21.3, Xp22.33-p21.3, Xq21.32-q21.33	1p22.2-q21.1, 1q21.1, 1q41-q44, 1q42.2, 1q44, 2p25.3-p25.2, 2p16.3, 2q14.3-q21.2, 3p14.2, 4p16.3-q35.2, 4q13.2, 4q22.1, 4q34.3, 6p22.1, 6p22.1-p21.33, 6q12-q13, 6q14.1-q27, 6q26, 7q11.22, 7q31.1, 7q31.33-q36.1, 8p21.3, 8q11.21-q21.3, 8q11.21, 8q13.2, 9p24.3-q33.3, 9p23, 9p21.3, 9p21.1, 10p15.3-p14, 10q25.3, 11p15.5-p14.3, 11q11, 11q14.1, 11q22.1-q23.2, 11q22.2, 11q23.3, 12p13.33-p13.2, 12q21.2-q24.21, 16p13.3, 16p13.3-p13.2, 16q23.1-q24.3, 16q23.1, 17p13.3, 17p12, 18q12.3-q22.2, 18q12.3-q21.1, 18q21.1, 18q21.1-q21.32, 18q21.32-q22.2, 19p13.3-p13.2, 20p13-p11.22, 20p12.1, 20q13.3, 22q11.1-q13.33, Xp21.3-q25

TMK-1	1p36.33-p36.11, 1p32.3, 1p11.2-q43, 1q21.2-q23.1, 1q25.1-q25.2, 1q32.1, 1q42.2-q43, 1q43, 2q33.1-q33.2, 5p15.33-q11.1, 5q31.2-q31.3, 6p21.32-p21.1, 7p11.2-q21.11, 7q21.3-q22.3, 8q22.1-q22.3, 8q24.21, 9p21.1-q34.3, 9q33.2-q34.13, 10p15.3-q11.21, 10q23.33-q25.1, 12q13.11-q13.13, 14q11.2-q24.3, 14q13.1-q13.2, 14q32.11-q32.12, 15q13-q26.2, 15q15.1-q21.1, 16p13.3-q24.3, 16q22.1-q22.2, 17p11.1-q25.3, 18p11.32-q12.2, 18p11.21-q11.2, 19q13.3-q13.43, 19q13.11, 19q13.11-q13.12, 20q11.21-q13.33, 20q11.21-q11.23, 20q13.32-q13.33	1p36.11-p35.3, 1q21.3, 1q24.3-q25.1, 1q42.13-q42.2, 1q43-q44, 2p25.3-q37.3, 2p16.3-p16.2, 2p12-p11.2, 2q21.3-q22.3, 2q33.3-q37.3, 3p24.1, 3p14.2, 3q26.1, 4p16.3-q28.2, 4p14-p13, 4q21.21, 4q34.3, 5q34-q35.3, 6p22.1-p21.33, 6q11-q27, 6q11.1-q12, 7q31.33-q32.1, 7q36.1, 7q36.3, 8p23.3-p22, 8p22, 8p11.23-p11.22, 9p23, 9p21.3, 10q21.1-q23.33, 10q21.1, 10q23.31, 11p15.5-p14.2, 11p15.5-p15.4, 12p13.33-p13.31, 12p13.31-p11.21,12q24.31-q24.32, 13q12.11-q34, 13q12.11-q14.3, 13q14.3-q31.3, 14q31.1-q31.3, 14q32.2-q32.33, 17q22, 18q12.3-q23, 18q22.3-q23, 19p13.3, 20p12.1, 20p12.1-p11.21, 21q11.2-q22.3, 22q11.23, Xq25-q26.1
