# Supplementary material for: Genome-wide gene copy number and expression analysis of primary gastric tumors and gastric cancer cell lines
Source: BMC Cancer. 2010 Mar 1;10:73. doi: 10.1186/1471-2407-10-73 (PMC2837868; doi:10.1186/1471-2407-10-73)
Supplement: Additional file 3 — Copy number associated gene expression changes. [file 1471-2407-10-73-S3.PDF]

Additional file 3. Copy number associated gene expression changes.

GeneName	Description	Fold change	Region
ALPK2	alpha-kinase 2	-34,6	18q12.3-q22.2
SETBP1	SET binding protein 1	-13,1	18q12.3-q22.2
CCDC102B	coiled-coil domain containing 102B	-5,6	18q12.3-q22.2
CD274	CD274 molecule	-4,4	9p24.3-p21.1
NFIB	nuclear factor I/B	-4,4	9p24.3-p21.1
SERPINB3	serpin peptidase inhibitor, clade B (ovalbumin), member 3	-4,3	18q12.3-q22.2
SERPINB4	serpin peptidase inhibitor, clade B (ovalbumin), member 4	-4,0	18q12.3-q22.2
KATNAL2	katanin p60 subunit A-like 2	-3,7	18q12.3-q22.2
MOBKL2B	MOB1, Mps One Binder kinase activator-like 2B (yeast)	-3,3	9p24.3-p21.1
AK3	adenylate kinase 3	-3,0	9p24.3-p21.1
SMARCA2	SWI/SNF related, matrix associated, actin dependent regulator of chromatin, subfamily a, member 2	-2,9	9p24.3-p21.1
MLANA	melan-A	-2,7	9p24.3-p21.1
SERPINB5	serpin peptidase inhibitor, clade B (ovalbumin), member 5	-2,7	18q12.3-q22.2
RCL1	RNA terminal phosphate cyclase-like 1	-2,7	9p24.3-p21.1
TSHZ1	teashirt zinc finger homeobox 1	-2,6	18q22.3-qter
LRRC19	leucine rich repeat containing 19	-2,6	9p24.3-p21.1
JAK2	Janus kinase 2 (a protein tyrosine kinase)	-2,6	9p24.3-p21.1
KIAA0427	KIAA0427	-2,4	18q12.3-q22.2
CCDC68	coiled-coil domain containing 68	-2,3	18q12.3-q22.2
INSL4	insulin-like 4 (placenta)	-2,3	9p24.3-p21.1
HSPA13	heat shock protein 70kDa family, member 13	-2,3	21q11.2-q21.1
TPD52L3	tumor protein D52-like 3	-2,3	9p24.3-p21.1
SERPINB8	serpin peptidase inhibitor, clade B (ovalbumin), member 8	-2,3	18q12.3-q22.2
MTAP	methylthioadenosine phosphorylase	-2,2	9p24.3-p21.1
SERPINB2	serpin peptidase inhibitor, clade B (ovalbumin), member 2	-2,2	18q12.3-q22.2
PMAIP1	phorbol-12-myristate-13-acetate-induced protein 1	-2,2	18q12.3-q22.2
TUSC1	tumor suppressor candidate 1	-2,1	9p24.3-p21.1
SMAD7	SMAD family member 7	-2,1	18q12.3-q22.2
JMJD2C	jumonji domain containing 2C	-2,1	9p24.3-p21.1
WDR7	WD repeat domain 7	-2,0	18q12.3-q22.2
ZNF578	zinc finger protein 578	2,0	19q12-qter
VGF	VGF nerve growth factor inducible	2,0	7q21.3-q11.1
LGALS8	lectin, galactoside-binding, soluble, 8	2,0	1q41-q43.1
ZNF320	zinc finger protein 320	2,0	19q12-qter
ZNF416	zinc finger protein 416	2,0	19q12-qter
RBM34	RNA binding motif protein 34	2,0	1q41-q43.1
LILRA6	leukocyte immunoglobulin-like receptor, subfamily A (with TM domain), member 6	2,0	19q12-qter
GUK1	guanylate kinase 1	2,0	1q41-q43.1
PARD6B	par-6 partitioning defective 6 homolog beta (C. elegans)	2,0	20p13-qter
CEP250	centrosomal protein 250kDa	2,0	20p13-qter
GML	glycosylphosphatidylinositol anchored molecule like protein	2,0	8q24.3
ZNF223	zinc finger protein 223	2,0	19q12-qter
COL22A1	collagen, type XXII, alpha 1	2,0	8q24.13-q24.3
NALP12	NLR family, pyrin domain containing 12	2,0	19q12-qter
TSKS	testis-specific kinase substrate	2,1	19q12-qter
CAPN2	calpain 2, (m/II) large subunit	2,1	1q41-q43.1
TNNT1	troponin T type 1	2,1	19q12-qter
AP1G2	adaptor-related protein complex 1, gamma 2 subunit	2,1	14q11.2
ARV1	ARV1 homolog (S. cerevisiae)	2,1	1q41-q43.1
RIN2	Ras and Rab interactor 2	2,1	20p13-qter
SGK2	serum/glucocorticoid regulated kinase 2	2,1	20p13-qter
DDX27	DEAD (Asp-Glu-Ala-Asp) box polypeptide 27	2,1	20p13-qter
OSMR	oncostatin M receptor	2,1	5p13.3-q11.1
ZNF552	zinc finger protein 552	2,1	19q12-qter
SPAG4L	sperm associated antigen 4-like	2,1	20p13-qter
NIPBL	Nipped-B homolog (Drosophila)	2,1	5p13.3-q11.1
LEFTY2	left-right determination factor 2	2,1	1q41-q43.1
CHGB	chromogranin B (secretogranin 1)	2,1	20p13-qter
RNASEN	ribonuclease type III, nuclear	2,1	5p13.3-q11.1
DNAJC21	DnaJ (Hsp40) homolog, subfamily C, member 21	2,1	5p13.3-q11.1
RAI14	retinoic acid induced 14	2,1	5p13.3-q11.1
CPNE1	copine I	2,1	20p13-qter
IFT52	intraflagellar transport 52 homolog (Chlamydomonas)	2,1	20p13-qter
DDEF1IT1	DDEF1 intronic transcript 1 (non-protein coding)	2,1	8q24.13-q24.3
ZNF586	zinc finger protein 586	2,1	19q12-qter
FUT1	fucosyltransferase 1 (galactoside 2-alpha-L-fucosyltransferase, H blood group)	2,1	19q12-qter
PSENEN	presenilin enhancer 2 homolog (C. elegans)	2,1	19q12-qter
MED25	mediator complex subunit 25	2,1	19q12-qter
LTB4R	leukotriene B4 receptor	2,1	14q11.2
FRG1B	FSHD region gene 1 family, member B	2,1	20p13-qter
TM9SF1	transmembrane 9 superfamily member 1	2,1	14q11.2
GOLPH3	golgi phosphoprotein 3 (coat-protein)	2,1	5p13.3-q11.1
CST5	cystatin D	2,1	20p13-qter
PTPRA	protein tyrosine phosphatase, receptor type, A	2,1	20p13-qter
MGC35440	hypothetical protein MGC35440	2,1	19q12-qter
NLRP2	NLR family, pyrin domain containing 2	2,1	19q12-qter
ZNF543	zinc finger protein 543	2,1	19q12-qter
TOMM20	translocase of outer mitochondrial membrane 20 homolog (yeast)	2,1	1q41-q43.1
U2AF1L4	U2 small nuclear RNA auxiliary factor 1-like 4	2,1	19q12-qter
ZNF570	zinc finger protein 570	2,2	19q12-qter
SIPA1L2	signal-induced proliferation-associated 1 like 2	2,2	1q41-q43.1
CEACAM5	carcinoembryonic antigen-related cell adhesion molecule 5	2,2	19q12-qter
KLK1	kallikrein 1	2,2	19q12-qter
PLEKHG2	pleckstrin homology domain containing, family G (with RhoGef domain) member 2	2,2	19q12-qter
ATP4A	ATPase, H+/K+ exchanging, alpha polypeptide	2,2	19q12-qter
ZNF565	zinc finger protein 565	2,2	19q12-qter
BLCAP	bladder cancer associated protein	2,2	20p13-qter
LSM14A	LSM14A, SCD6 homolog A (S. cerevisiae)	2,2	19q12-qter
MEIS3	Meis homeobox 3	2,2	19q12-qter
GGPS1	geranylgeranyl diphosphate synthase 1	2,2	1q41-q43.1
DEFB123	defensin, beta 123	2,2	20p13-qter
ID1	inhibitor of DNA binding 1, dominant negative helix-loop-helix protein	2,2	20p13-qter

TASP1	taspase, threonine aspartase, 1	2,2	20p13-qter
ZNF137	zinc finger protein 137	2,2	19q12-qter
RRBP1	ribosome binding protein 1 homolog 180kDa (dog)	2,2	20p13-qter
PAIP1	poly(A) binding protein interacting protein 1	2,2	5p13.3-q11.1
FLJ40243	hypothetical protein FLJ40243	2,2	5p13.3-q11.1
RPL37	ribosomal protein L37	2,2	5p13.3-q11.1
ZNF444	zinc finger protein 444	2,2	19q12-qter
ZNF667	zinc finger protein 667	2,2	19q12-qter
CDC42BPA	CDC42 binding protein kinase alpha (DMPK-like)	2,2	1q41-q43.1
IPO4	importin 4	2,2	14q11.2
ZNF284	zinc finger protein 284	2,3	19q12-qter
ZSWIM1	zinc finger, SWIM-type containing 1	2,3	20p13-qter
RPS16	ribosomal protein S16	2,3	19q12-qter
UGT3A2	UDP glycosyltransferase 3 family, polypeptide A2	2,3	5p13.3-q11.1
LILRA2	leukocyte immunoglobulin-like receptor, subfamily A (with TM domain), member 2	2,3	19q12-qter
ASAP1	ArfGAP with SH3 domain, ankyrin repeat and PH domain 1	2,3	8q24.13-q24.3
ZNF616	zinc finger protein 616	2,3	19q12-qter
ZSWIM3	zinc finger, SWIM-type containing 3	2,3	20p13-qter
KCNS1	potassium voltage-gated channel, delayed-rectifier, subfamily S, member 1	2,4	20p13-qter
UBA2	ubiquitin-like modifier activating enzyme 2	2,4	19q12-qter
TSNAX	translin-associated factor X	2,4	1q41-q43.1
ZNF813	zinc finger protein 813	2,4	19q12-qter
FLRT3	fibronectin leucine rich transmembrane protein 3	2,4	20p13-qter
SNX5	sorting nexin 5	2,4	20p13-qter
ERF	Ets2 repressor factor	2,4	19q12-qter
ZNF226	zinc finger protein 226	2,4	19q12-qter
BMP7	bone morphogenetic protein 7	2,5	20p13-qter
CRLS1	cardiolipin synthase 1	2,5	20p13-qter
GPR4	G protein-coupled receptor 4	2,5	19q12-qter
ZNF331	zinc finger protein 331	2,5	19q12-qter
RNF43	ring finger protein 43	2,5	17q22-q24.2
NOVA2	neuro-oncological ventral antigen 2	2,5	19q12-qter
RAD1	RAD1 homolog (S. pombe)	2,5	5p13.3-q11.1
FAM83D	family with sequence similarity 83, member D	2,5	20p13-qter
LYPD2	LY6/PLAUR domain containing 2	2,5	8q24.3
CNTD2	cyclin N-terminal domain containing 2	2,5	19q12-qter
SIPA1L3	signal-induced proliferation-associated 1 like 3	2,5	19q12-qter
MARK4	MAP/microtubule affinity-regulating kinase 4	2,5	19q12-qter
ZNF773	zinc finger protein 773	2,5	19q12-qter
HAO1	hydroxyacid oxidase (glycolate oxidase) 1	2,5	20p13-qter
VRK3	vaccinia related kinase 3	2,5	19q12-qter
LGI4	leucine-rich repeat LGI family, member 4	2,6	19q12-qter
ZNF571	zinc finger protein 571	2,6	19q12-qter
YIF1B	Yip1 interacting factor homolog B (S. cerevisiae)	2,6	19q12-qter
DKKL1	dickkopf-like 1 (soggy)	2,6	19q12-qter
SLC13A3	solute carrier family 13 (sodium-dependent dicarboxylate transporter), member 3	2,6	20p13-qter
AZGP1	alpha-2-glycoprotein 1, zinc-binding	2,6	7q21.3-q11.1
ZNF613	zinc finger protein 613	2,6	19q12-qter
GNPAT	glyceronephosphate O-acyltransferase	2,7	1q41-q43.1
ZNF134	zinc finger protein 134	2,7	19q12-qter
ZNF28	zinc finger protein 28	2,7	19q12-qter
LY6H	lymphocyte antigen 6 complex, locus H	2,7	8q24.13-q24.3
ZNF550	zinc finger protein 550	2,7	19q12-qter
HCST	hematopoietic cell signal transducer	2,7	19q12-qter
APCDD1L	adenomatosis polyposis coli down-regulated 1-like	2,7	20p13-qter
KCNN4	potassium intermediate/small conductance calcium-activated channel, subfamily N, member 4	2,7	19q12-qter
B3GALNT2	beta-1,3-N-acetylgalactosaminyltransferase 2	2,7	1q41-q43.1
SLC35F3	solute carrier family 35, member F3	2,8	1q41-q43.1
TGM2	transglutaminase 2 (C polypeptide, protein-glutamine-gamma-glutamyltransferase)	2,8	20p13-qter
XRN2	5'-3' exoribonuclease 2	2,8	20p13-qter
ZNF461	zinc finger protein 461	2,8	19q12-qter
CST3	cystatin C	2,8	20p13-qter
ZNF614	zinc finger protein 614	2,8	19q12-qter
LTBP4	latent transforming growth factor beta binding protein 4	2,8	19q12-qter
ABHD12	abhydrolase domain containing 12	2,8	20p13-qter
FLJ30596	chromosome 5 open reading frame 33	2,9	5p13.3-q11.1
FBXO28	F-box protein 28	2,9	1q41-q43.1
ZNF131	zinc finger protein 131	2,9	5p13.3-q11.1
CD40	CD40 molecule, TNF receptor superfamily member 5	2,9	20p13-qter
MAP1LC3A	microtubule-associated protein 1 light chain 3 alpha	3,0	20p13-qter
SDCBP2	syndecan binding protein (syntenin) 2	3,0	20p13-qter
CCNE1	cyclin E1	3,0	19q12-qter
KIAA0355	KIAA0355	3,0	19q12-qter
DUSP5P	dual specificity phosphatase 5 pseudogene	3,0	1q41-q43.1
HEATR1	HEAT repeat containing 1	3,0	1q41-q43.1
TTYH1	tweety homolog 1 (Drosophila)	3,0	19q12-qter
TYROBP	TYRO protein tyrosine kinase binding protein	3,0	19q12-qter
ZNF404	zinc finger protein 404	3,1	19q12-qter
TARBP1	TAR (HIV-1) RNA binding protein 1	3,1	1q41-q43.1
CSRP2BP	CSRP2 binding protein	3,1	20p13-qter
CST2	cystatin SA	3,2	20p13-qter
RNF14	ring finger protein 114	3,2	20p13-qter
CYP2F1	cytochrome P450, family 2, subfamily F, polypeptide 1	3,2	19q12-qter
APOC1	apolipoprotein C-I	3,2	19q12-qter
EYA2	eyes absent homolog 2 (Drosophila)	3,2	20p13-qter
PDZD2	PDZ domain containing 2	3,2	5p13.3-q11.1
SLC7A9	solute carrier family 7 (cationic amino acid transporter, y+ system), member 9	3,3	19q12-qter
MGC42105	hypothetical protein MGC42105	3,3	5p13.3-q11.1
TGM1	transglutaminase 1 (K polypeptide epidermal type I, protein-glutamine-gamma-glutamyltransferase)	3,3	14q11.2
MOSC1	MOCO sulphurase C-terminal domain containing 1	3,4	1q41-q43.1
TUBB1	tubulin, beta 1	3,4	20p13-qter
HIF3A	hypoxia inducible factor 3, alpha subunit	3,4	19q12-qter
CLC	Charcot-Leyden crystal protein	3,4	19q12-qter
KIAA1804	mixed lineage kinase 4	3,4	1q41-q43.1
HARS2	histidyl-tRNA synthetase 2, mitochondrial (putative)	3,5	20p13-qter

FLJ32363	chromosome 5 open reading frame 34	3,5	5p13.3-q11.1
IL4I1	interleukin 4 induced 1	3,5	19q12-qter
GGT7	gamma-glutamyltransferase 7	3,5	20p13-qter
LYPD3	LY6/PLAUR domain containing 3	3,6	19q12-qter
ZFP14	zinc finger protein 14 homolog (mouse)	3,6	19q12-qter
PI3	peptidase inhibitor 3, skin-derived	3,6	20p13-qter
DHDH	dihydrodiol dehydrogenase (dimeric)	3,7	19q12-qter
AXIN2	axin 2	3,7	17q22-q24.2
DEGS1	degenerative spermatocyte homolog 1, lipid desaturase (Drosophila)	3,7	1q41-q43.1
PPP1R1B	protein phosphatase 1, regulatory (inhibitor) subunit 1B	3,7	17q12-q21.1
ZNF334	zinc finger protein 334	3,8	20p13-qter
TARS	threonyl-tRNA synthetase	3,8	5p13.3-q11.1
ZNF600	zinc finger protein 600	3,8	19q12-qter
FTL	ferritin, light polypeptide	3,8	19q12-qter
DEFB126	defensin, beta 126	3,8	20p13-qter
ZNF615	zinc finger protein 615	3,9	19q12-qter
ZNF529	zinc finger protein 529	3,9	19q12-qter
EGFLAM	EGF-like, fibronectin type III and laminin G domains	3,9	5p13.3-q11.1
AGT	angiotensinogen (serpin peptidase inhibitor, clade A, member 8)	4,0	1q41-q43.1
TTC9B	tetratricopeptide repeat domain 9B	4,1	19q12-qter
PLCB1	phospholipase C, beta 1 (phosphoinositide-specific)	4,1	20p13-qter
FLJ12355	hypothetical protein FLJ12355	4,1	19q12-qter
KHDRBS3	KH domain containing, RNA binding, signal transduction associated 3	4,2	8q24.13-q24.3
ZFP82	zinc finger protein 82 homolog (mouse)	4,2	19q12-qter
ENAH	enabled homolog (Drosophila)	4,2	1q41-q43.1
OXCT1	3-oxoacid CoA transferase 1	4,3	5p13.3-q11.1
WDR26	WD repeat domain 26	4,3	1q41-q43.1
BAI1	brain-specific angiogenesis inhibitor 1	4,3	8q24.13-q24.3
HPN	hepsin (transmembrane protease, serine 1)	4,5	19q12-qter
CYP3A4	cytochrome P450, family 3, subfamily A, polypeptide 4	4,6	7q21.3-q11.1
NLRP7	NLR family, pyrin domain containing 7	4,7	19q12-qter
APOE	apolipoprotein E	4,7	19q12-qter
ERBB2	v-erb-b2 erythroblastic leukemia viral oncogene homolog 2	5,2	17q12-q21.1
ZNF256	zinc finger protein 256	5,3	19q12-qter
SLC1A3	solute carrier family 1 (glial high affinity glutamate transporter), member 3	5,6	5p13.3-q11.1
ZNF671	zinc finger protein 671	5,6	19q12-qter
LIFR	leukemia inhibitory factor receptor alpha	5,6	5p13.3-q11.1
PSG2	pregnancy specific beta-1-glycoprotein 2	5,7	19q12-qter
ZNF606	zinc finger protein 606	5,9	19q12-qter
FLJ13231	chromosome 5 open reading frame 42	6,0	5p13.3-q11.1
ZNF480	zinc finger protein 480	6,1	19q12-qter
DACT3	dapper, antagonist of beta-catenin, homolog 3 (Xenopus laevis)	6,2	19q12-qter
VN1R1	vomeronasal 1 receptor 1	6,3	19q12-qter
CAPSL	calcyphosine-like	6,3	5p13.3-q11.1
MARK1	MAP/microtubule affinity-regulating kinase 1	6,4	1q41-q43.1
ZNF71	zinc finger protein 71	6,5	19q12-qter
CST1	cystatin SN	6,5	20p13-qter
CYP3A7	cytochrome P450, family 3, subfamily A, polypeptide 7	6,6	7q21.3-q11.1
TMEM145	transmembrane protein 145	6,6	19q12-qter
ZNF432	zinc finger protein 432	6,7	19q12-qter
LRRC6	leucine rich repeat containing 6	6,8	8q24.13-q24.3
CSTL1	cystatin-like 1	6,9	20p13-qter
ZNF701	zinc finger protein 701	7,0	19q12-qter
WNT3A	wingless-type MMTV integration site family, member 3A	7,1	1q41-q43.1
TBX4	T-box 4	7,3	17q22-q24.2
MMP9	matrix metallopeptidase 9	7,3	20p13-qter
ZNF772	zinc finger protein 772	7,4	19q12-qter
KLK12	kallikrein-related peptidase 12	8,5	19q12-qter
ICAM2	intercellular adhesion molecule 2	9,0	17q22-q24.2
FPR1	formyl peptide receptor 1	9,3	19q12-qter
ZNF114	zinc finger protein 114	9,5	19q12-qter
SULF2	sulfatase 2	10,4	20p13-qter
HSPA12B	heat shock 70kD protein 12B	10,7	20p13-qter
CEACAM7	carcinoembryonic antigen-related cell adhesion molecule 7	11,3	19q12-qter
PHACTR3	phosphatase and actin regulator 3	14,4	20p13-qter
ZNF577	zinc finger protein 577	14,8	19q12-qter
ZIK1	zinc finger protein interacting with K protein 1 homolog (mouse)	18,1	19q12-qter
SLC7A10	solute carrier family 7, (neutral amino acid transporter, y+ system) member 10	23,8	19q12-qter
HHIPL2	HHIP-like 2	26,9	1q41-q43.1

Gene expression fold change = (median expression of cancer samples with copy number alteration)/
(median gene expression of cancer samples with normal copy number).
